# Othram maps: a graph-powered platform for pedigree visualization and forensic intelligence

**DOI:** 10.1093/bioinformatics/btag047

**Published:** 2026-01-24

**Authors:** Stephen Newman, Bruce Budowle, Kristen Mittelman, David Mittelman

**Affiliations:** Othram Inc., The Woodlands, TX 77381, United States; Othram Inc., The Woodlands, TX 77381, United States; Department of Forensic Medicine, University of Helsinki, Helsinki, Finland; Forensic Science Institute, Radford University, Radford, VA 24013, United States; Othram Inc., The Woodlands, TX 77381, United States; Othram Inc., The Woodlands, TX 77381, United States

## Abstract

**Motivation:**

Forensic genetic genealogy (FGG) requires building and interpreting complex family trees that integrate genetic, genealogical, and contextual metadata to associate unknown donors of forensic evidence with reference samples of potential relatives. Existing genealogy platforms are not optimized for forensic workflows, lack scalable infrastructure for large pedigree trees, and pose usability challenges in investigative settings. *Othram Maps* is a graph-powered platform for generating, ranking, and visualizing genealogical hypotheses. It was developed to support forensic investigations but also has applications in adjacent domains such as biomedical research and genetic epidemiology.

**Results:**

*Othram Maps* is a graph-powered web platform designed for forensic intelligence applications. Unlike traditional tree tools, it uses a flexible graph architecture that enables dynamic visualization and exploration of family trees of any size or structure encountered in forensic genetic genealogy. A custom load-on-demand engine supports smooth navigation of trees with thousands of individuals, while global and detailed views provide scalable, real-time interactivity for collaborative casework. Othram Maps is actively used by genetic genealogists and forensic practitioners to identify human remains and investigate unsolved violent crimes.

**Availability and implementation:**

*Othram Maps* is freely available at https://maps.othram.com. The platform is fully web-based and runs on all modern desktop and mobile browsers without the need for installation. It is compatible with most current mobile devices, enabling secure, on-the-go access for field or collaborative work. Additional information on the platform’s features, functionality, and algorithms is provided in the Methods, available as supplementary data at *Bioinformatics* online.

## 1 Introduction

Forensic genetic genealogy (FGG) is a powerful method for human identification that combines DNA analysis with genealogical records to infer identity or kinship (Dowdeswell 2022, Mandape *et al.* 2024, Budowle *et al.* 2025). In many cases, investigators must construct, interpret, and refine complex family trees that span multiple generations that may contain hundreds to thousands of individuals. These trees often integrate diverse sources of information, including autosomal DNA matches, genealogical documents, and investigative metadata. Despite the central role of FGG workflows, there are few purpose-built tools to support their use in forensic investigations.

Most existing family tree software tools are tailored to consumer genealogy or academic pedigree analysis (Shan and Luther 2025). These systems tend to assume clean, well-documented family relationships, offer limited support for metadata-driven hypotheses, and typically cannot scale to the sizes required for modern long-range kinship analyses. Additionally, many are not optimized for collaborative, browser-based use.

*Othram Maps* was developed to address these limitations by providing four core functions. They are: (i) a Genealogical Relationship Calculator, which estimates the possible relationship(s) of any two people in a tree, (ii) the Genetic Genealogical Positioning System (GGPS) which analyzes DNA matches and genealogical data to point where an unknown person likely fits in a tree, (iii) a Most Recent Common Ancestor (MRCA) Path Builder, which builds a visual pathway of connecting multiple individuals to their most recent common ancestors, and (iv) and a Surname Connector Finder, which analyzes multiple subtrees to identify shared or similar surnames to reveal potential relationships between or among families.

Current approaches to tree building, relationship visualization, and kinship assessment in forensic genetic genealogy typically rely on flat, hierarchical, or tiered pedigree representations. These structures require substantial manual effort, are prone to missing potential connections due to user-driven interfacing, and do not scale efficiently to large datasets. In contrast, GGPS is a graph-powered platform that generates, ranks, and visualizes genealogical hypotheses for forensic investigations. By leveraging data structures optimized for relational analysis, GGPS efficiently models simple to complex kinship networks involving hundreds or thousands of individuals. Rather than manually tracing relationships, users can query the graph to discover and prioritize potential connections, accelerating analysis and reducing reliance on individual expertise.

## 2 Features and implementation

*Othram Maps* is a web-based platform for scalable, interactive visualization and analysis of genealogical trees. It models individuals and relationships as nodes and edges in a directed graph rather than as rigid hierarchical trees. This graph-based architecture accommodates incomplete or ambiguous relationships common in real-world genealogy and enables efficient manipulation and traversal of large networks.

### 2.1 Genetic genealogical positioning system (GGPS)

GGPS is a graph-based analytical framework that automates the generation, ranking, and visualization of genealogical hypotheses from DNA match data. Designed for forensic genetic genealogy, GGPS systematically explores all plausible ancestral paths linking an unknown individual to known relatives, quantifies the likelihood and effort associated with each path, and prioritizes connections most consistent with the observed DNA evidence. In essence, it operationalizes the process of genealogical reasoning, transforming what was once an expert-driven, manual task into a reproducible and data-driven search for the most probable position of an unknown individual within a family network.

Ertürk et al. (2022) described a formal mathematical treatment of the genealogy process used in FGG. These authors recast forensic genetic genealogy as a stochastic dynamic program that chooses, at each step, whether to ascend (investigate a match), descend (from a candidate MRCA list), or stop, using a cost-effectiveness objective that balances probability of success against expected workload. This framework formalized the manual genealogical process of balancing the likelihood of finding the right family line against the time and effort needed to explore it. However, two limitations of this model are that it operates in an abstract, simulated space reasoning about probabilities as opposed to actual people or families and was designed to study the genealogical process in principle rather than to generate actionable hypotheses for specific cases.

DNA Painter’s *What Are the Odds?* (WATO) is another popular web-based tool that helps genealogists estimate where an unknown individual might fit within a provided family tree based on shared centimorgans (cM). The tool evaluates hypotheses proposed by the user and reports relative odds for each possible placement within the descendants of a most recent common ancestor (MRCA). While intuitive and accessible, WATO requires users to define the MRCA in advance and does not automatically explore the broader search space. It does not identify points of convergence among multiple matches or address the substantial manual effort required to construct and test hypotheses across large or complex pedigrees.

**Figure 1:**
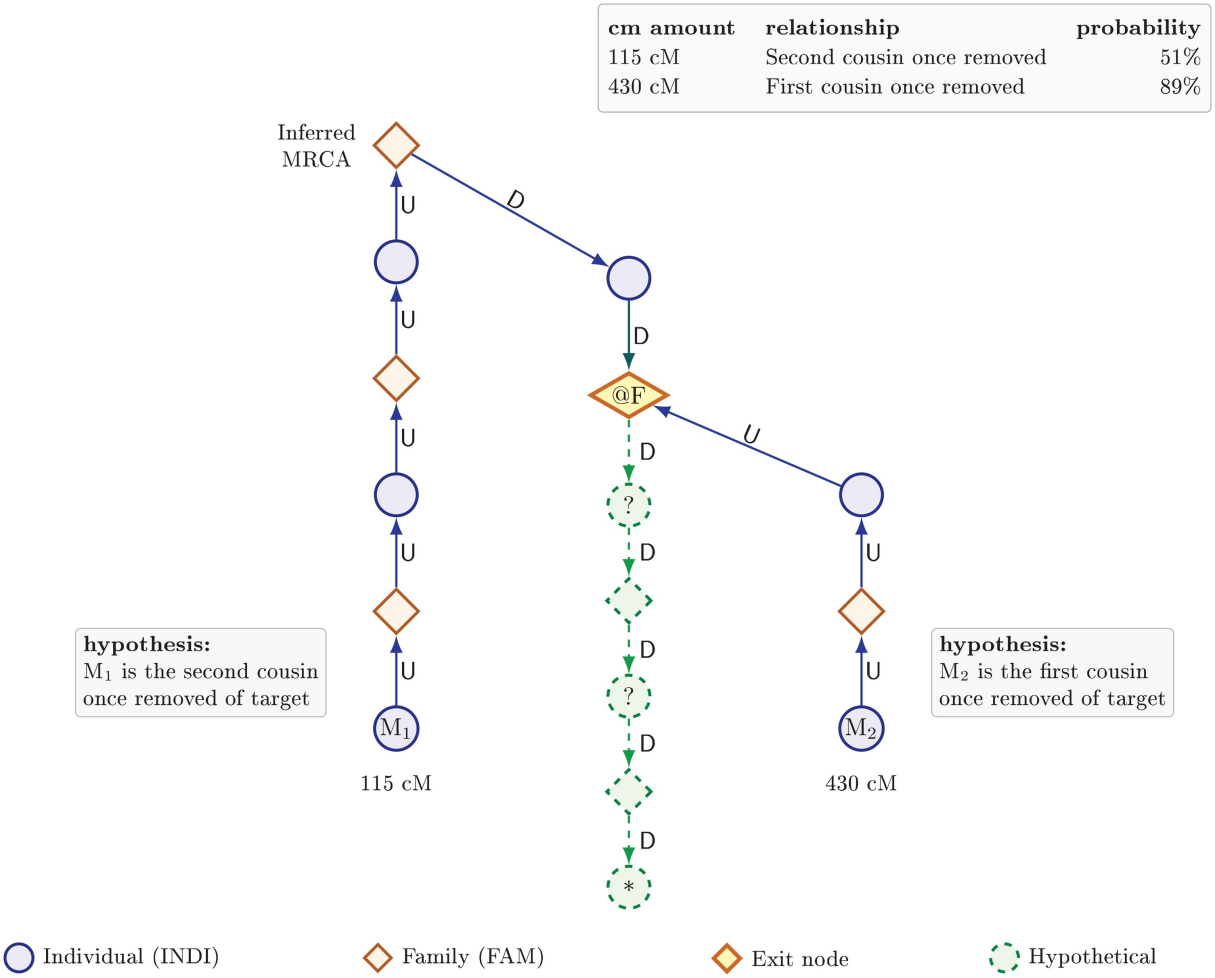
Example GGPS hypothesis visualization. Two matches (M and M) connect to the target (*) through distinct ancestral paths that converge at a family node (@F). Each path forms a testable hypothesis, in this case M as a second cousin once removed (115 cM, 51%) and M as a rst cousin once removed (430 cM, 89%). Green nodes represent hypothetical individuals required to complete the path, contributing to the workload term in the GGPS ranking score.

GGPS combines the analytical insight of Ertürk’s probabilistic framework with the practical cM-based approach of WATO, and extends both into an operational, data-driven system. Built on a graph database of individuals and families, GGPS automatically traces all plausible genealogical paths (using “up” and “down” movements through generations) for each DNA match and then detects where those paths intersect across multiple matches. Each intersection represents a concrete, testable hypothesis about where the unknown person could fit in the family structure. These hypotheses are then ranked using a unified workload-probability score that captures both the effort required to explore each branch and the combined DNA evidence supporting it (Fig. 1).

GGPS implements an end-to-end, graph-driven hypothesis generator and ranker. On a Neo4j genealogical graph with INDI↔FAM nodes and granular U/D movement, verified patterns are traced for each match and exit-node intersections are detected where different matches converge on the same node v with the same remainingMoves r. Each (v, r) is a testable hypothesis. Paths are ranked with a unified “lower-is-better” score: (W_eff + α)/(P_combined · P_t · Q_factor), where W_eff is genealogical workload with partial-generation penalties, P_t = qa^g_up · qd^g_down, P_combined is the geometric mean of per-match cM probabilities, and Q_factor rewards probability consistency. Age range and minimum per relationship probability filters help suppress low-quality hypotheses. Users are not required to pre-select a single MRCA couple; GGPS can surface hypotheses anywhere in the connected graph, and disconnected subtrees are handled as separate components when selecting matches (for more detail see Methods, available as supplementary data at *Bioinformatics* online).

GGPS improves on approaches described above by turning the conceptual models of Ertürk *et al.* and WATO into an integrated positioning algorithm, akin to a GPS for genetic genealogy. It replaces dynamic-program thresholds over simulated states with concrete graph traversal and intersection detection over real people and families and discovers graph-wide convergence rather than evaluating placements only within a provided MRCA-scoped subtree. Instead of instructing the user how to drive (as Ertürk et al. 2022) or asking the user to suggest possible destinations (as WATO does), GGPS analyzes the full map of relationships and signals where the unknown person most likely belongs in the tree. Critically, GGPS explicitly aggregates multi-match cM evidence for each path via a geometric mean (with a quality factor), models half/bidirectional relationships and partial moves, and returns remainingMoves r that translate directly into “what to build next.” Execution is server-side and scalable, with interpretable, reproducible ranking tied to actual graph structure. By grounding probabilistic reasoning in a real genealogical graph, GGPS transforms a theoretical framework into an operational system capable of efficiently and reproducibly locating individuals within complex family networks.

### 2.2 Core functionality and analysis tools

*Othram Maps* includes a range of features to support complex genealogical analysis and investigative workflows. Users can interactively pan, zoom, and reposition nodes to explore subtrees and relationships, with metadata such as names, birthdates, and inferred familial links accessible through intuitive hover and click actions.

For large trees, the platform offers a Global View that activates when the field of view exceeds 500 nodes. This view is generated by subdividing the coordinate space into grid-based tiles and running optimized graph queries in Neo4j to aggregate individuals and families within each tile. Density metrics, including counts, total entities, and geometric centers, are calculated, along with representative sample coordinates for precise navigation. This tile-based aggregation allows seamless zooming between detailed and global views while maintaining full spatial continuity across the same coordinate system.

Investigators can annotate individual nodes with case notes and metadata fields, integrating contextual information directly into the visual workspace. The search functionality allows users to quickly locate individuals by name or identifier and highlight them within a tree.

Users can select any two individuals and request a genealogical relationship path through the Genealogical Relationship Calculator. In cases of pedigree collapse or endogamy, where multiple connections may exist, the platform traverses the graph and returns all valid relationship paths. This capability is essential for interpreting complex kinship in populations with dense ancestral overlap. The relationship identification process begins with common-ancestor discovery within a configurable depth limit. Upward paths from both individuals are traced until intersection points (ie, shared ancestors) are identified. These intersections are then used to reconstruct the path between individuals via bidirectional traversal, generating both the shortest-path relationship and any additional valid connections.

Building on this foundation, the MRCA Path Builder visually reconstructs how multiple individuals connect to shared ancestors, enabling investigators to see and document the full lineage pathway that supports a hypothesized relationship. The resulting diagram can be exported as a high-resolution, printable figure suitable for inclusion in judicial proceedings-ready forensic reports.

Finally, the Surname Connector Finder analyzes multiple subtrees (or disconnected trees) to detect shared or similar surnames, revealing potential cross-family links or latent connections between clusters. This tool assists in early hypothesis generation by highlighting possible overlaps that merit further investigation.

Each node, whether representing an individual or a family unit, offers access to a detailed data view. For users requiring deeper visibility, a developer mode can be activated to inspect the raw GEDCOM source underlying a given node. This feature facilitates transparency, troubleshooting, and precise validation of imported genealogical records.

To protect personal privacy, particularly in shared or published trees, Othram Maps includes a redaction feature that automatically hides individuals likely to be living. Even without explicit birth or death dates, living status is inferred from generational position, proximity to known deceased relatives, and other contextual graph data. A graph-based mortality inference algorithm analyzes up to five generations of descendants using Neo4j’s variable-length path matching; if a descendant aged 85 or older is identified, the ancestor is inferred to be deceased. Names of living individuals can be redacted from visualizations while underlying data remain available for secure search and analytical inference.

To build trees, *Othram Maps* employs an approach that adapts to complexity and structure. The platform currently uses an incremental-force algorithm by default. This hybrid algorithm operates in distinct phases: initially, it identifies connected components within the family graph to process isolated family groups independently, then applies positioning using a hierarchical graph layout algorithm to establish proper generational ordering with parents positioned above children and families. For large components containing more than 150 nodes, the system implements a two-phase approach where family nodes are initially positioned using massive spacing parameters (600 px horizontal, 2000 px vertical) to prevent overlap, followed by positioning individuals around these family anchor points. The algorithm then refines these positions using a force-directed simulation that applies collision avoidance, link constraints, and centering forces while preserving the established hierarchy.

### 2.3 GEDCOM support and interoperability

*Othram Maps* is fully compatible with GEDCOM, the long-standing standard format for genealogical data exchange (Family History Department 2019). GEDCOM (GEnealogical Data COMmunication), first introduced by the Church of Jesus Christ of Latter-day Saints in the 1980s, has become the *de facto* format for exchanging genealogical information between software platforms. However, in practice, different tools and versions often implement the standard inconsistently, leading to compatibility issues.

To address this issue, *Othram Maps* includes a highly tolerant GEDCOM parser capable of reading the most common versions in use today. Once ingested, the data are normalized and written back out in a clean, standardized GEDCOM 5.5.1 format. This capability allows users to correct inconsistencies, clean noisy datasets, and export them in a reproducible form for archival or external analysis.

Uniquely, *Othram Maps* extends GEDCOM by encoding layout-specific metadata within the file, including the graph layout coordinates, last viewed zoom level, DNA matches, viewport position, viewing history and bookmarks. This capability means that when a GEDCOM file is saved from the platform and later reloaded, even in a different session or by another user, it restores the full workspace context, including visual configuration. These enhancements maintain backward compatibility and preserve interoperability with other tools.

### 2.4 System architecture and platform capability

*Othram Maps* is implemented as a single-page web application with a cloud-hosted backend. All visualization occurs in the browser, providing zero-install access across desktop and mobile devices. The platform features a custom-built, dynamic “load-on-demand” rendering engine that fetches and displays tree segments in real time, ensuring responsive performance even for very large genealogical networks.

The backend combines PostgreSQL for operational data (users, access control, organizations) with daily backups, and a Neo4j graph database for storing family trees. This design enables efficient traversal and graph-based algorithms such as lowest-common-ancestor (LCA) discovery, multi-path detection, and half-relationship identification.

*Othram Maps* is accessible through all modern web browsers, including Chrome, Firefox, Safari, and mobile browsers. The platform is fully responsive and optimized for performance across desktop and mobile devices, including tablets and smartphones.

## 3 Discussions and future work

*Othram Maps* was developed to address a core bottleneck in FGG: the scalable, interpretable visualization of family structures derived from complex and often incomplete data. By moving beyond traditional tree representations and adopting a graph-based model, the platform enables flexible handling of real-world genealogical ambiguity, such as unknown parentage, endogamy, or repeated individuals. This architecture also allows for forensic-specific features like match-based overlays and inference of missing relatives, which are essential in long-range identification workflows.

While the platform is designed with forensic use cases in mind, its capabilities are broadly applicable to adjacent domains that require exploration of complex pedigree structures such as genetic epidemiology, population genetics, and clinical genetics (e.g. rare disease segregation). The ability to integrate metadata, annotate nodes, and dynamically traverse large trees would support uses in both research and applied settings.

*Othram Maps* is actively maintained and iteratively improved based on feedback from forensic practitioners, genealogists, and researchers. As the field of FGG continues to evolve, it is anticipated that platforms like *Othram Maps* will play a central role in making complex family structures more navigable, explainable, and actionable.

## Supplementary Material

btag047_Supplementary_Data

## References

[btag047-B3] Budowle B , Mittelman K, Mittelman D. Genomics will forever reshape forensic science and criminal justice. Genome Biol 2025;26:296. 40983944 10.1186/s13059-025-03798-x PMC12455754

[btag047-B6] Dowdeswell TL. Forensic genetic genealogy: a profile of cases solved. Forensic Sci Int Genet 2022;58:102679. 35176668 10.1016/j.fsigen.2022.102679

[btag047-B7] Ertürk MS , Fitzpatrick C, Press M et al Analysis of the genealogy process in forensic genetic genealogy. J Forensic Sci 2022;67:2218–29. 36059116 10.1111/1556-4029.15127 PMC9826014

[btag047-B8] Family History Department, T.C.o.J.C.o.L.-d.S. *The GEDCOM Standard: Release 5.5.1*. Salt Lake City, UT: The Church of Jesus Christ of Latter-day Saints; 2019. https://gedcom.io/specifications/ged551.pdf

[btag047-B10] Mandape SN , Budowle B, Mittelman K et al Dense single nucleotide polymorphism testing revolutionizes scope and degree of certainty for source attribution in forensic investigations. Croat Med J 2024;65:249–60. 38868971 10.3325/cmj.2024.65.249 PMC11157251

[btag047-B11] Shan F , Luther K. Reexamining technological support for genealogy research, collaboration, and education. Proc ACM Hum Comput Interact 2025;9:1–33.40909183

