# Supplementary material for: Othram maps: a graph-powered platform for pedigree visualization and forensic intelligence
Source: Bioinformatics. 2026 Jan 24;42(2):btag047. doi: 10.1093/bioinformatics/btag047 (PMC12883083; doi:10.1093/bioinformatics/btag047)
Supplement: btag047_Supplementary_Data [file btag047_supplementary_data.pdf]

Supplementary Methods

GGPS: Genetic Genealogical Positioning for Intersection-Based Autosomal DNA Hypotheses

A graph-driven method is developed that generates and prioritizes relationship hypotheses based on autosomal DNA across multiple DNA matches. The system computes candidate relationship paths for each match, detects full intersections across matches at shared exit nodes with identical remaining paths and ranks hypotheses using a unified workload_probability score.

1. Graph database: Neo4j stores individuals and families with parent_child and reference relationships.

2. Granular U/D paths: Relationships are modeled as strings over {U, D} denoting genealogical moves up (U) and down (D). Verified granular patterns (including bidirectional variants and half-relationships) are provided by a pattern service.

3. cM data: Total shared centimorgans (cM) per match are evaluated against Shared cM Project distributions to obtain per-relationship probabilities.

Granular U/D tracing and node semantics

1. The tree is modeled with two node types: Individual (INDI) and Family (FAM). Every individual is connected to one or more family nodes (birth family; one family per union), and individuals are never directly connected to other individuals. Genealogical steps always alternate between INDI and FAM nodes.

2. Moves and constraints: The U/D notation represents each U (up) or D (down) as a single genealogical step oriented toward ancestry (U) or descent (D) while traversing the alternating INDI ↔ FAM structure. Traces obey a simple-path constraint which is the same node in a single path is not revisited.

Examples (see https://maps.othram.com)

1. Sibling (UD): start at the proband (INDI), go U to the proband's birth family (FAM), then D to the sibling (INDI).
2. Half-sibling (UUDD): start at the proband (INDI), go U to birth family (FAM), U to the parent (INDI), then D to the parent's other family (FAM), and D to the half-sibling (INDI). Importantly, we never move back to the same family node; the descent proceeds via a distinct family node.
3. Bidirectionality: some relationship classes are bidirectional, meaning they admit two verified granular patterns obtained by swapping the counts of U and D moves relative to perspective (e.g., 1c1r has patterns like 3U/5D and 5U/3D). Other relationships are inherently directional and are modeled as distinct types rather than a single bidirectional class (e.g., aunt/uncle versus niece/nephew).

Candidate relationship generation (per match)

1. Infer a candidate set of relationship types from the match's cM value.
2. For each relationship type, enumerate its verified granular U/D pattern(s).
3. Execute an efficient, two-phase traversal: ascend U moves from the proband to candidate ancestors, then descend D moves to candidate targets. Each traversal yields completedMoves, remainingMoves, and an exit node (family or individual) where traversal paused with remainingMoves.

Intersection-based hypothesis formation (multi-match)

1. For N matches, exit nodes are collected across all candidate relationships and identify full intersections where all N matches converge on the same exit node with the same remainingMoves pattern.
2. This definition is independent of the starting relationship type. Each intersection contains per-path hypotheses (one per contributing match/path) and an aggregated exit-node hypothesis.

Intersection formalization

Let $G = (V_I \cup V_F, E)$ be a bipartite directed graph with individual nodes V_I, family nodes V_F, and edges $E \subseteq (V_I \times V_F) \cup (V_F \times V_I)$ (no individual_individual edges). Let $\Sigma = \{U, D\}$ be the move alphabet and define a pattern map $\pi : \Sigma^* \rightarrow P$ that returns the set of alternating paths in G consistent with a movement string (one edge per symbol; two moves per generation).

Fix a target individual $T \in V_I$. For a match i, relationship tracing over all tested types produces a set of exit annotations

$$E_i \subseteq (V_I \cup V_F) \times \Sigma^*, \ (v, r) \in E_i \Longleftrightarrow v \text{ is an exit node for } i \text{ with remaining pattern } r.$$

For a selected set of matches S, the set of full-intersection keys is

$$I_S = \{ (v, r) \in (V_I \cup V_F) \times \Sigma^* : \forall i \in S, (v, r) \in E_i \}.$$

Each $(v, r) \in I_S$ defines a hypothesis $H = (v, r, S, \text{paths}(v, r))$, where paths(v, r) collects per-match path details and evidence. Scoring S(H) is given by the unified workload_probability formula in the next section and is computed using R.

Exit nodes and hypothetical paths

An exit node is the point where traversal pauses with a residual U/D sequence remainingMoves).
Exit nodes can be either individual (INDI) nodes or family (FAM) nodes.

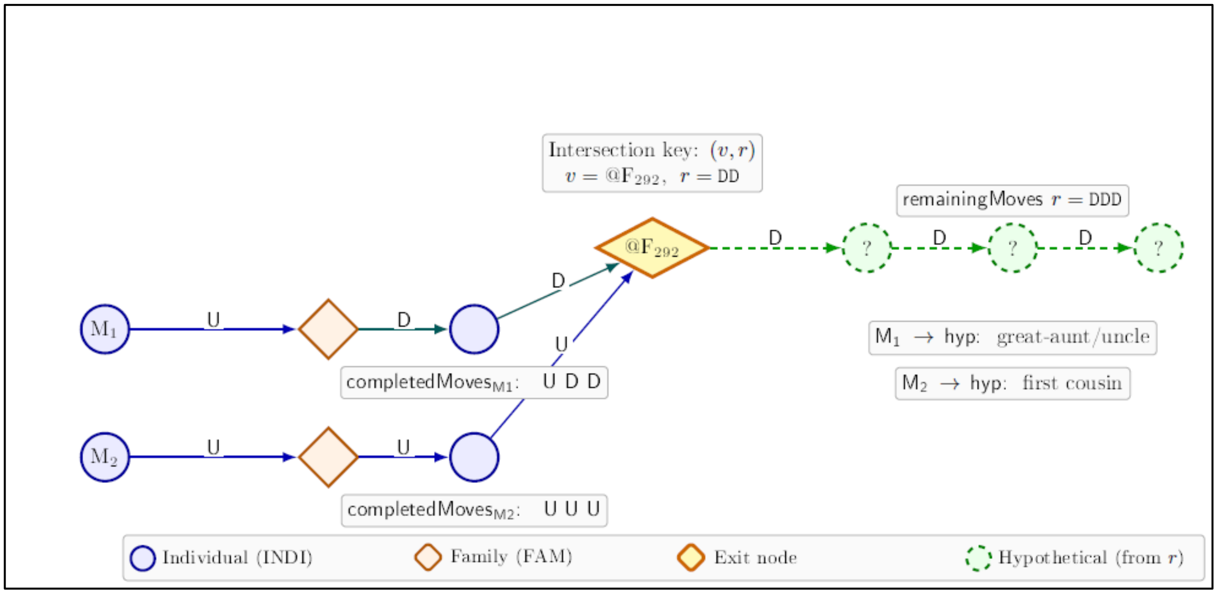

Supplementary Figure 1: Intersection-based hypothesis generation and ranking. Two matches (M1, M2) traverse U/D steps to the same exit node (e.g., family @F292) with the same remaining path r = DDD. The remaining moves (dashed) lead to hypothetical targets. A full intersection occurs when all selected

matches share the same (v, r). Hypotheses are ranked by a unified score combining workload, combined cM evidence, and transition success.

Self vs. Hypothetical
If the potential unknown individual resolves to an actual individual already present in the tree, the relationship is labeled self . Otherwise, the residual U/D sequence defines a hypothetical path that terminates in a hypothetical individual (not yet in the tree). Building out this hypothetical path provides concrete candidates to investigate. Once added and documented, these candidates can be validated against the cM evidence and other matches.

Interpretation
For each exit node, the report lists contributing individuals and hypothesized relationships they would have to the exit-node individual if the remainingMoves were completed as specified. Each hypothesis enumerates relationships with respect to the exit node: self denotes the individual named in the exit node; child denotes a child of the exit node; grandchild, great-grandchild, etc., follow analogously (and likewise parent, grandparent, etc.). This structure supports third-party record collection and incremental hypothesis testing.

Unified workload_probability scoring (lower = better)
We rank per-path and aggregated exit-node hypotheses with a unified score that reflects exactly what the implementation computes:

$$\text{SCORE} = \frac{W_{\text{eff}} + \alpha}{P_{\text{combined}}\, P_t\, Q_{\text{factor}}}.$$

Movement pattern to generations and partials
Let remainingMoves contain U and D moves. Define u = #U, d = #D, gup = ⌊u/2⌋, gdown = ⌊d/2⌋, and partials uodd = u mod 2, dodd = d mod 2 (each generation requires two moves).
Workload (expected people to investigate).

Full-generation ascent and descent workloads are

$$N_a = 2^{g_{up}+1} - 2, \qquad N_d = n_g \frac{n_g^{g_{down}} - 1}{n_g - 1}, \qquad n_g = 2.5.$$

where ng is the average number of children per family with children. An ng = 2.5 is adopted as a default; Ertürk et al. estimate ng from historical fertility data (and it can vary by generation in their modeling).

Partial-generation penalty (from odd moves):

$$W_{\text{partial}} = 1.0\, u_{\text{odd}} + 1.25\, d_{\text{odd}}.$$

Combined cM evidence (geometric mean). Given per-individual probabilities in percent P%i, first discard any non-positive values, then convert to decimals and clamp via pi = max(MIN_PROB, min(1, P%_i/100)), with MIN_PROB = 0.001.

The combined probability is the geometric mean

$$P_{\text{combined}} = \left(\prod_{i=1}^{n} p_i\right)^{1/n}.$$

Transition success
The probability of successfully identifying people along the path is

$$P_t = q_a^{g_{up}}\, q_d^{g_{down}}, \qquad q_a = 0.60,\ q_d = 0.98.$$

Where qa is the per-generation probability of successfully identifying parents (ascent), and qd is the per-generation probability of successfully identifying children (descent). Values of qa = 0.60 and qd = 0.98 are adopted directly from Ertürk et al. (2022, Table 2) and are treated as fixed constants in the scoring (not estimated from our data).

Quality factor
Consistency and sufficiently high individual probabilities are rewarded:

$$\text{mean} = \frac{1}{n}\sum_i p_i, \qquad \text{cv} = \frac{\sqrt{\frac{1}{n}\sum_i (p_i - \text{mean})^2}}{\text{mean}};$$

$$\text{consistency} = \max\big(1.0,\ \min(1.2,\ 1.2 - 0.5\,\text{cv})\big);$$

$$\text{hqRatio} = \frac{\#\{i : p_i \geq 0.10\}}{n}, \qquad \text{qualityBonus} = 1.0 + 0.3\,\text{hqRatio};$$

$$Q_{\text{factor}} = \min\big(1.5,\ \text{consistency} \times \text{qualityBonus}\big).$$

Constants
α = 1.0 is a stability (Laplace-style) addended to the workload. It adds one notional person to the numerator so that extremely low-work paths (e.g., near-complete or partial-move cases) do not score unrealistically well when probabilities are high. It has minimal impact on medium/large workloads but regularizes small-work regimes. Wmin = 0.1 is used as a minimum workload floor and MIN_PROB = 0.001 to clamp very small probabilities for 'complete' relationships where the workload would have been zero.

Aggregated exit-node hypotheses use the best constituent path score (minimum) with capped boosts for convergence and hypothesis diversity. Groups never rank worse than their strongest member.

Relationship translation and interpretability
Remaining U/D patterns are translated to readable relationship names (e.g., DDDDDD → great-grandchild), enabling clear interpretation of what remains to complete a hypothesis from a given exit node.

Tree setup and usage workflow
1. Navigate to https://maps.othram.com.

2. Load or build a tree: upload a GEDCOM, or construct a tree using right-click actions (add individuals/families). A demo tree with pre-entered cM is available for exploration.

3. Enter cM values: click an individual, choose Edit in the right sidebar, enter the total shared cM, and Save. Use the search (magnifying-glass) in the right sidebar to quickly locate people.

4. Open Relationship tools (right sidebar) and select GGPS. The panel lists all individuals with cM values, grouped by connected component (disconnected subtrees). Hypotheses operate only within a single connected component. Check/uncheck individuals from the same component to choose the set for the run. If no intersections appear, try a different subset; a lack of results can also indicate a placement error or pedigree collapse.

5. Advanced options: optionally set an age range for the target individual and a minimum per-relationship probability threshold (from Shared cM data) to filter hypotheses.

6. Generate: press ‘Generate Hypotheses’. Processing typically takes 15 seconds to several minutes depending on tree size/complexity. Results are returned as cards (one per exit node), each summarizing one or more converging paths/hypotheses along with the relationship between the exit node (person named on the card) and potential unknown (i.e. if it is 'self' then the exit node is a potential unknown, if it is 'child' then the child of the exit node individual is the potential unknown.)
